# Neuropathological Similarities and Differences between Schizophrenia and Bipolar Disorder: A Flow Cytometric Postmortem Brain Study

**DOI:** 10.1371/journal.pone.0033019

**Published:** 2012-03-15

**Authors:** Yoshitaka Hayashi, Naomi Nihonmatsu-Kikuchi, Shin-ichi Hisanaga, Xiu-jun Yu, Yoshitaka Tatebayashi

**Affiliations:** 1 Affective Disorders Research Team, Tokyo Metropolitan Institute of Medical Science, Tokyo, Japan; 2 Department of Biological Sciences, Graduate School of Science, Tokyo Metropolitan University, Tokyo, Japan; 3 Department of Neurology, Key Laboratory of Neurology of Hebei Province, Second Hospital of Hebei Medical University, Shijiazhuang, China; Rikagaku Kenkyūsho Brain Science Institute, Japan

## Abstract

Recent studies suggest that schizophrenia (SCH) and bipolar disorder (BPD) may share a similar etiopathology. However, their precise neuropathological natures have rarely been characterized in a comprehensive and quantitative fashion. We have recently developed a rapid, quantitative cell-counting method for frozen unfixed postmortem brains using a flow cytometer. In the present study, we not only counted stained nuclei, but also measured their sizes in the gray matter of frontopolar cortices (FPCs) and inferior temporal cortices (ITCs) from patients with SCH or BPD, as well as in that from normal controls. In terms of NeuN(+) neuronal nuclei size, particularly in the reduced densities of small NeuN(+) nuclei, we found abnormal distributions present in the ITC gray matter of both patient groups. These same abnormalities were also found in the FPCs of SCH patients, whereas in the FPCs of BPD patients, a reduction in oligodendrocyte lineage (olig2(+)) cells was much more common. Surprisingly, in the SCH FPC, normal left-greater-than-right asymmetry in neural nuclei densities was almost completely reversed. In the BPD FPC, this asymmetry, though not obvious, differed significantly from that in the SCH FPC. These findings indicate that while similar neuropathological abnormalities are shared by patients with SCH or BPD, differences also exist, mainly in the FPC, which may at least partially explain the differences observed in many aspects in these disorders.

## Introduction

The similarities and differences between schizophrenia (SCH) and bipolar disorder (BPD) have long been of interest among a wide range of research fields in psychiatry. Recent epidemiological and genetic findings suggest that SCH and BPD have certain common etiological factors, or share several chromosomal loci and genes, which may confer vulnerability to these disorders [1]-[3]. In neuroimaging studies, however, the results have proven somewhat mixed. For example, the most consistent gross anatomical changes found in SCH include lateral and third ventricular enlargement, medial temporal lobe volume reduction, and superior temporal cortex volume reduction, particularly on the left side [4]. In BPD, however, mild ventricular enlargement and the presence of white matter hyper-intensities remain among the most consistently reported abnormalities [5]. Such evidence gives rise to several important questions regarding psychiatric neuropathology: (i) To what extent do the phenotypic expressions of these disorders overlap? (ii) Where and how do these disorders diverge? (iii) What are the neuropathological changes associated with these gross anatomical abnormalities?

Although a few definitive quantitative neuropathological findings for either SCH and/or BPD have been described with regards to the temporal cortex, several histopathological studies examining the prefrontal cortex have consistently reported the potential role of interneurons in the pathophysiology of SCH and BPD. For example, using Nissl-stained sections, Benes et al [6]. were the first to provide evidence that SCH patients had reduced numbers of small non-pyramidal interneurons in layers I and II, as well as increased numbers of pyramidal neurons in layer V of the frontopolar cortex (FPC; BA 10), without any concomitant total neuronal loss. Subsequent studies of the SCH prefrontal cortex have almost consistently supported their results, demonstrating the presence of reduced gene expression levels for the 67 kDa isoform of glutamic acid decarboxylase (GAD67) [6]-[11], gamma-aminobutyric acid (GABA) membrane transporter 1 [12], reelin [8], [9], [11], and parvalbumin [10], as well as lower densities of neurons positive for GAD67 [13], parvalbumin [14], reelin [8], or calbindin [15]. Furthermore, some of these alterations were also observed in the BPD (and in some cases of affective psychosis) prefrontal cortex [6], [8], [13], [15].

**Figure 1:**
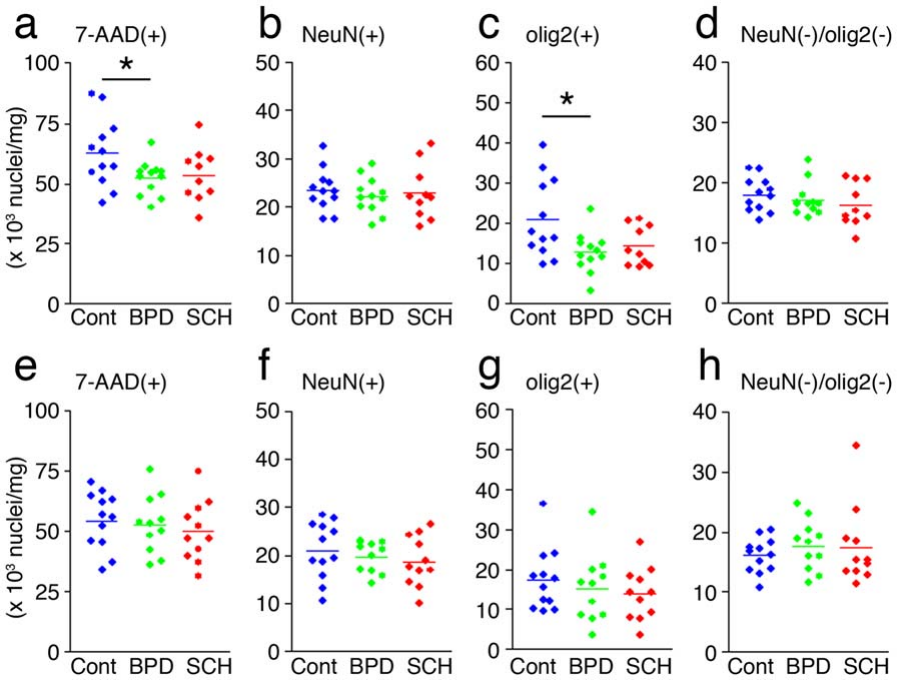
Densities of nuclei in 1 mg of gray matter tissue. (a-d) FPC and (e-h) ITC. (a, e) 7-AAD(+), (b, f) NeuN(+), (c, g) olig2(+), and (d, h) NeuN(−)/olig2(−) nuclei. *P<0.05 by unpaired *t*-test.

Recently, we developed a novel flow cytometeric (FCM) cell-counting method to quantify the densities and fluorescent intensities not only of neural cell nuclei labeled for DNA (total), but also of the neuronal and oligodendroglial nuclear markers, 7-AAD, NeuN and olig2, respectively, in frozen unfixed postmortem brains [16]. In the present study, we applied this method to investigate the neuropathological similarities and differences in the gray matter of the FPC and ITC from patients with SCH or BPD, as well as from normal controls. Furthermore, while taking the utmost care for osmotic control, we measured nuclear size, which was particularly important in distinguishing those small NeuN(+) nuclei that roughly represent small non-pyramidal interneurons.

## Results

### FCM measurement of nuclei densities

We first quantified gray matter nuclei densities of frozen postmortem FPCs and ITCs from patients with BPD (FPC, n = 12; ITC, n = 11) and SCH (FPC, n = 10; ITC, n = 11), as well as from normal controls (FPC, n = 12; ITC, n = 12) obtained from the Stanley Foundation Neuropathological Consortium (Table S1). We found a significant decrease in the densities of 7-AAD(+) (total) nuclei in the FPCs of BPD subjects (−16%, *P* = 0.038), but not in SCH patients (*P* = 0.118) (Fig. 1a). No significant differences were found in the densities of NeuN(+) and NeuN(−)/olig2(−) nuclei in the FPCs of either group, nor in those containing total (7-AAD(+)), NeuN(+), olig2(+) and NeuN(−)/olig2(−) nuclei in the ITC of control versus BPD or SCH patients (Figure 1b,d,e-h).

**Figure 2:**
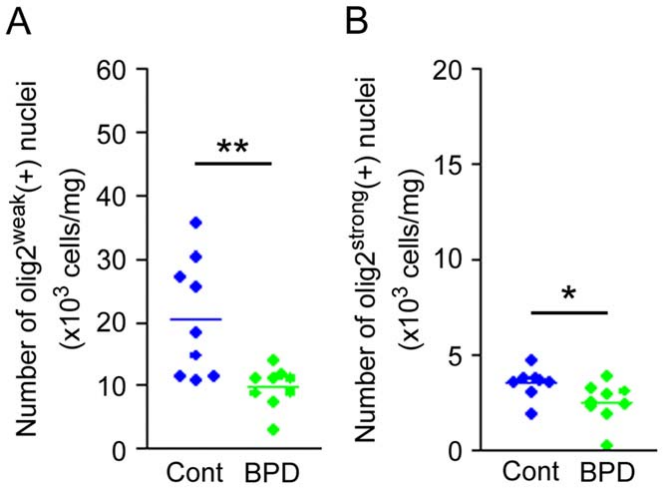
Densities of olig2^weak^(+) and olig2^strong^(+) nuclei in 1 mg of gray matter tissue from BPD FPC. (A) olig2^weak^(+), and (B) olig2^strong^(+) nuclei. *P<0.05 by unpaired *t*-test.

A significant group difference was found in the densities of olig2(+) nuclei in the FPCs of BPD patients (−40%, *P* = 0.018) (Figure 1c). In a subgroup of BPD FPC samples, in which two peaks of olig2(+) populations could be clearly measured (9 control, 9 BPD), significant decreases were also found in the densities of both olig2^strong^(+) (−28%, *P* = 0.034) and olig2^weak^(+) nuclei (−53%, *P* = 0.008) (Figure 2), suggesting that oligodendroglial reductions in the BPD FPC may stem from decreased levels of OPCs (olig2^strong^(+)) and OLs (olig2^weak^(+)), as was observed in the FPC from subjects with MDD [16].

### Validation of forward scatter (FS) values as nuclei size

**Figure 3:**
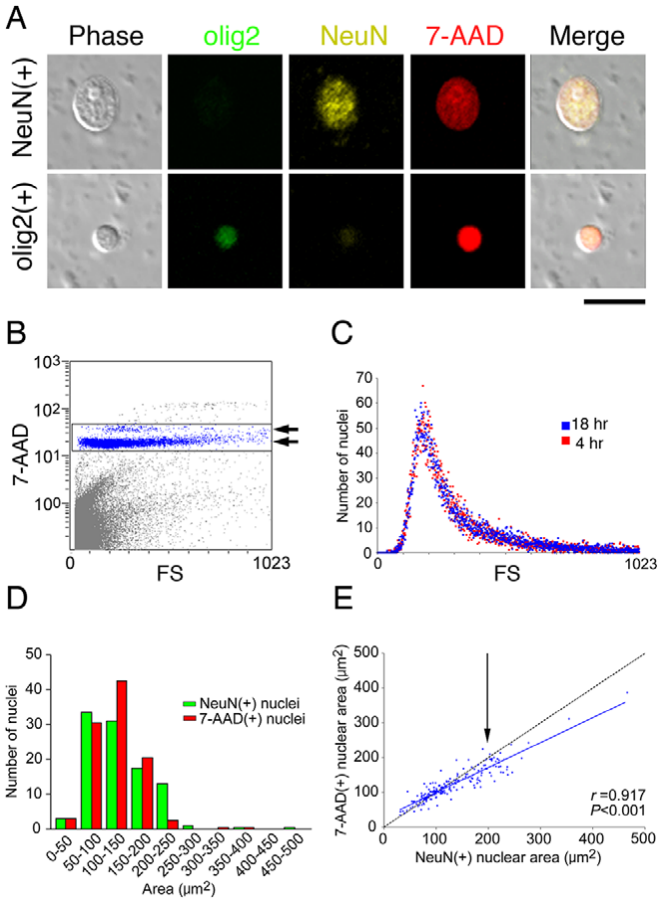
Validation of forward scatter (FS) values of nuclei isolated from frozen unfixed human postmortem brains. (A) Appearance of a human NeuN(+) (upper) or olig2(+) nucleus (lower) in PBS (280 mOsm). Scale bar, 20 µm. (B) FCM measurement of 7-AAD(+) nuclei. Arrows indicate two major populations of 7-AAD(+) nuclei. (C) Stability of isolated NeuN(+) nuclei in PBS. No difference was found in the FS distributions of NeuN(+) nuclei analyzed at 4 h and 18 h after homogenization, suggesting that the size of NeuN(+) nuclei remained quite stable in PBS. (D) Distribution of 7-AAD(+) (red) and NeuN(+) (green) nuclei areas (µ m^2^) as determined by microscopic measurements. The nuclei populations were delineated every 50 µm^2^. (E) Correlation between the areas of 7-AAD(+) and NeuN(+) nuclei (µ m^2^) as measured microscopically. The arrow (200 µm^2^ of NeuN(+) area) indicates a point that corresponds approximately to FS 400. Beginning with this point, the area of the NeuN(+) nuclei became gradually larger than that of the 7-AAD(+) nuclei, although the areas of the 7-AAD(+) and NeuN(+) nuclei correlated to a significant degree (*r* = 0.917, *P*<0.001).

Forward scatter (FS) values (FS 0-1023) are generally associated with particle size in the context of FCM measurements [17]. It our experimental settings, however, it was unclear whether the FS values we obtained really represented nuclei size. We therefore first examined the appearance and stability of the isolated nuclei from frozen unfixed postmortem brains. In our experimental procedure, the osmotic pressures of nuclei suspensions were kept within the physiological range (280 ∼ 290 mOsm), except for the isolation process in which lysis buffer with ∼375 mOsm was used in order to protect nuclei from mechanical homogenization. Figure 3A shows the typical appearance of isolated human nuclei in PBS stained with NeuN or olig2. We found no leakage of DNA marker (7-AAD) from any isolated nuclei (Figure 3A, 7-AAD(+)), suggesting that the nuclear envelopes were well preserved in our isolation and staining processes. This was further supported by the FCM measurement of 7-AAD(+) nuclei, in which 7-AAD(+) nuclei were clearly aligned on the two horizontal lines with almost uniform florescent intensities (Figure 3B, arrows, the thin upper line is probably composed of nuclei in the G2 and M phases of the cell cycle), suggesting that full DNA components (sometimes double amounts of DNA) are stably preserved in the nuclei even during FCM measurement. We also examined the temporal stability of FS values. No difference was found in the distribution of FS values for any of the stained nuclei between 4 and 18 hours after homogenization (Figure 3C, NeuN), suggesting that the FS values of isolated nuclei were quite stable, at least during the experimental period.

We then compared the distribution patterns between FS values and nuclear size, measuring them microscopically (Figure S1). We found that the distribution of FS values of NeuN(+) nuclei almost matched NeuN(+) nuclei size (Figure 3D), suggesting that the FS values actually represent nuclear size. However, we noted that the isolation of large NeuN(+) nuclei (FS 400 ≥) remained incomplete, allowing NeuN(+) perinuclear structures to attach themselves around the true border of the nuclei (Figure 3E; for a more detailed view, see Figure S1c). Furthermore, preliminary animal studies revealed that PMIs (but not gender or storage period) significantly increased the FS values of NeuN(+) nuclei in a time-dependent manner, albeit without changing the total numbers of NeuN(+) nuclei or the peak heights of the NeuN(+) nuclei distribution pattern (Figure S2). These findings suggest that while the FS values of small NeuN(+) nuclei represent, in a relatively reliable fashion, nuclei size, those of large NeuN(+) nuclei (FS 400 ≥) may be inaccurate. We therefore excluded large NeuN(+) nuclei (FS 400 ≥) from further analyses in our human postmortem brain studies.

### FCM measurement of NeuN(+) nuclei size

**Figure 4:**
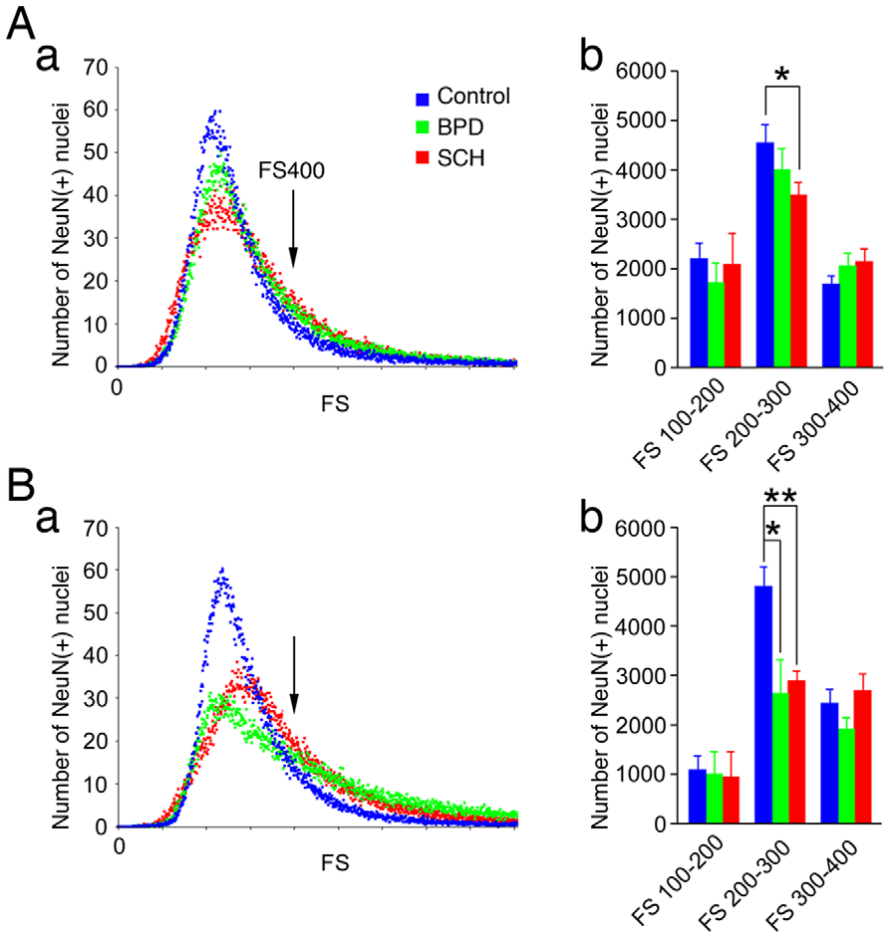
Distribution of FS values of NeuN(+) nuclei (10,000 nuclei) in the gray matter tissue. (A) FPC, (B) ITC, (a) Abnormal distributions of FS values of NeuN(+) nuclei. (b) Abnormal reductions of the small NeuN(+) nuclei counts (FS 200 – 300) in SCH and BPD. * *P*<0.05, **P<0.01, unpaired *t*-test. mean±s.e.m.

In the distribution of FS values for the adjusted population (10,000) of olig2(+) and NeuN(−)/olig2(−) nuclei, no significant group difference was noted in either brain region. In contrast, a significant group difference was detected in the FS distribution of NeuN(+) nuclei. We found that the distribution of small NeuN(+) nuclei (especially the numbers of NeuN(+) nuclei with FS values of 200 – 300) had decreased in both brain regions of SCH patients (Figure 4, Table S2). In BPD subjects, however, while the distribution of small NeuN(+) nuclei in the ITC was also significantly lower, no major difference was found in the FPC (Figure 4, Table S2).

### Effects of confounding factors on nuclei counts

To examine the effects of confounding factors, we first performed Pearson’s correlations between the nuclei counts and countable confounding factors. Significant positive correlations were found in some confounding factors with the NeuN(+), olig2(+), or NeuN(−)/olig2(−) nuclei densities (Table S3). Thus, ANCOVA was performed using these variables as covariates and no change was noted regarding the main effects of the densities of NeuN(+) (FPC; BPD, *P* = 0.508, SCH, *P = *0.112), olig2(+) (FPC; BPD, *P = *0.013, SCH, *P = *0.072; ITC; BPD, *P = *0.843, SCH, *P = *0.305), or NeuN(−)/olig2(−) nuclei (ITC; BPD, *P* = 0.179, SCH, *P* = 0.183), nor was any recorded with the other nuclei.

Certain countable confounding factors such as age at onset, duration of disease, and fluphenazine equivalent were analyzed separately with only the diseased groups (Table S4). In the FPC, we found a significant positive correlation between duration of disease and total, NeuN(+), or NeuN(−)/olig2(−) nuclei densities. Since reduced gray matter volume has also been reported in the prefrontal areas of BPD [18] and SCH [19], and is often negatively correlated with the duration of disease [19], these positive correlations in the FPC may be explained in part by the reduced gray matter volumes of the FPC, the result of longer disease durations, although there is no concomitant loss of NeuN(+) and NeuN(−)/olig2(−) cells.

Both the postmortem interval (PMI) and the refrigeration interval were significantly longer in the SCH group (Table S1). We therefore excluded some samples with extremely long PMIs (< 40 hrs) and refrigeration intervals (< 20 hrs) in order to better match these variables. We then reanalyzed the rest of the samples and obtained essentially the same results as regards the nuclei densities (Table S5) and the NeuN(+) nuclei distribution patterns (Table S6).

We also examined the effects of uncountable variables such as hemisphere, gender, and severity of alcohol and/or substance abuse. We found that abnormalities in the densities of 7-AAD(+) and olig2(+) nuclei in BPD and SCH FPCs were more pronounced in the left hemispheres (Table S7). On the other hand, no such effects were found in the ITCs of BPD and SCH patients (Table S8).

**Figure 5:**
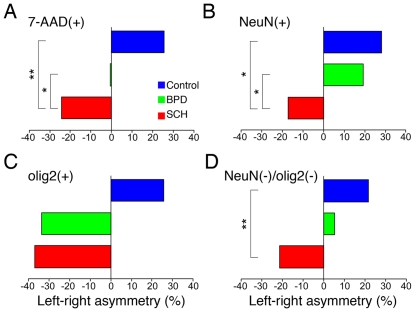
Asymmetry of the nuclei densities in the FPC. (A) 7-AAD(+), (B) NeuN(+), (C) olig2(+), and (D) NeuN(−)/olig2(−) nuclei. Left-right asymmetry rates (%) were calculated using the following formula: (the mean nuclei density in the left hemisphere)/(the mean nuclei density in the right hemisphere) ×100–100 (%). The positive % indicates left-greater-than-right asymmetry in the nuclei densities and the negative % vice versa. * *P*<0.05, **P<0.01, two-factor factorial ANOVA (diagnostic group×brain hemisphere).

Interestingly, in the control FPC, the left hemispheres tended to have increased densities of nuclei compared to the right hemispheres (Figure 5). Furthermore, these normal left-greater-than-right asymmetries were almost completely reversed in SCH subjects. Two-factor factorial ANOVA (diagnostic group×brain hemisphere) revealed significant group differences between control and SCH in the densities of 7-AAD(+) (F(1,18) = 8.93, *P* = 0.008), NeuN(+) (F(1,18) = 6.97, *P* = 0.017), NeuN(−)/olig2(−) (F(1,18) = 10.13, *P* = 0.005), but not in olig2 nuclei densities. In contrast, no significant group difference was found between control and BPD groups; rather, BPD differed from SCH in the 7-AAD (F(1,18) = 4.72, *P* = 0.043) and NeuN(+) nuclei densities (F(1,18) = 4.78, *P* = 0.042). In the ITC samples, no such clear left-right asymmetries or reversals in SCH patients were observed.

## Discussion

The present study applied a novel flow cytometric cell-counting method for frozen unfixed postmortem brain tissue as a means of investigating the neuropathological similarities and differences between SCH and BPD. While taking the utmost care for osmotic control, we measured the densities, fluorescence intensities, and FS values (sizes) of total (7-AAD(+)), neuronal (NeuN(+)), and oligodendroglial (olig2(+)) cell nuclei, as well as other neural cell (NeuN(−)/olig2(−)) nuclei in the gray matter of FPCs and ITCs from patients with either SCH or BPD, and from normal controls. We found that i) in the ITC, there exist abnormal reductions in the densities of small NeuN(+) nuclei, without a concomitant loss of total NeuN(+) nuclei densities, in both SCH and BPD; ii) in the FPC, by contrast, SCH was characterized primarily by the same abnormality noted in the densities of small NeuN(+) nuclei, whereas BPD was characterized by reductions in the densities of olig2(+) nuclei; iii) the normal left-greater-than-right asymmetry observed in the nuclei densities of control FPCs was almost completely reversed in those of SCH FPCs, albeit to a lesser extent than those of BPD FPCs; and iv) in terms of the 7-AAD(+) and NeuN(+) nuclei density asymmetries recorded in the FPC, there was a statistically significant group difference between SCH and BPD patients.

### Abnormalities in the inferior temporal cortex (BA20)

The densities of small NeuN(+) nuclei in the gray matter of ITC were abnormally reduced in both SCH and BPD patients without any concomitant loss of total NeuN(+) nuclei densities. To our knowledge, this is the first quantitative neuropathological study of the ITC from subjects with psychiatric disorders, suggesting that the densities of neurons with small NeuN(+) nuclei may be lower in the ITC of subjects with SCH or BPD. Since a high degree of correlation exists between nuclei size and perikaryon volume in neocortical neurons [20], small NeuN(+) nuclei may, in fact, be small non-pyramidal interneurons. There are reports of interneuronal abnormalities in widespread regions of SCH and/or BPD brains, including the dorsolateral prefrontal (DLPFC, BA9) [7], [8], [10], [12], anterior cingulate (ACC, BA24) [10], [13], [15], primary motor, and visual cortices [10], as well as in the hippocampus [9]. Our findings herein support, albeit in a different way, the following conclusions: i) in the SCH and BPD ITC, similar reductions in neurons with small NeuN(+) nuclei, which most likely are interneurons, were present; and ii) lower densities of small NeuN(+) neurons appeared to be compensated for by insignificant increases in the densities of large NeuN(+) neurons, without concomitant reductions of total NeuN(+) neurons.

Recent neuroimaging studies have shown that bilaterally smaller ITC volumes and/or cortical thinning are present in first-episode (as well as chronic) SCH and/or first-episode affective psychosis [21], [22], suggesting that certain neuropathological abnormalities exist in the ITC at the onset of illness, possibly even long before the onset. Our findings support this hypothesis, albeit in a different manner, and suggest that abnormally reduced small NeuN(+) neurons may constitute one of the neuropathological correlates associated with such neuroimaging abnormalities. Because the abnormal distribution pattern of small NeuN(+) nuclei in SCH differed slightly from that of BPD (Figure 4Ba), future studies will need to utilize different nuclear markers for interneurons, such as calcium-binding proteins and/or transcription factors, in order to more effectively distinguish the subtypes of interneurons and to more clearly characterize the neuropathological similarities and differences between SCH and BPD.

### Abnormalities in the frontopolar cortex (BA10)

In the SCH FPC, although not in the BPD FPC, we detected abnormal reductions in the densities of small NeuN(+) nuclei without any concomitant decrease in total NeuN(+) nuclei densities. Previous neuropathological studies of fixed brains have demonstrated similar neuronal abnormalities in the SCH FPC, albeit in different layers; i.e., reduced numbers (or densities) of small Nissl-stained neurons in layers I and II [6], parvalbumin-immunoreactive neurons in layers III and IV [14], and reelin-immunoreactive neurons in layers I, II, and IV [11]. Although our data does not address lamina formation, our findings are quite similar to those reported by Benes et al. [6], who, uniquely, utilized a vibratome instead of conventional paraffin sectioning for cutting the tissues. This was done in order to avoid the approximate 30% tissue shrinkage that occurs as a result of alcohol and xylene treatment during the paraffin-embedding process [6].

In the BPD FPC, in contrast, we observed reductions primarily in the densities of olig2(+) nuclei. Similar oligodendroglial abnormalities were also found in the FPC from subjects with major depressive disorder (MDD) [16], suggesting that cortical oligodendroglial abnormalities in the FPC, such as deficits in the proliferation and maturation of oligodendrocyte lineage (olig2(+)) cells, may represent a neuropathology common to various mood disorders. Interestingly, adulthood cortical myelination, an essential process for establishing an efficient neuronal network in the cortex [23], continues at least until the fifth decade in the FPC [24]. Further supporting our findings, myelin basic protein expression was reduced in the postmortem FPC of patients suffering from mood disorders, but not in those with SCH [25]. In neuroimaging studies, the presence of hyper-intensities is among the most consistently reported abnormality, not only in BPD but also in MDD brains [5], [26]. Future studies will be required to determine whether or not such oligodendroglial abnormalities are specific to mood disorders, or if they are common to psychiatric disorders in general, and simply exist to a greater extent in mood disorders than in SCH.

The FPC is the single largest architectonic region in the prefrontal cortex (PFC) that is almost exclusively connected to the more posterior supramodal areas such as the DLPFC and the orbitofrontal cortex [27]. Recent functional neuroimaging studies have revealed that the FPC probably plays roles in overcoming the limitations of more posterior prefrontal processes–such as working memory in the DLPFC [28], and decision making and hedonic processes in the OFC [29]–by enabling the emergence of more flexible, long-lasting, and possibly more rewarding cognitive control mechanisms as a human being seeks optimal social behavior [30].

### Hemispheric abnormalities in the frontopolar cortex (BA 10)

In the FPCs of control cases we found that the densities of nuclei in the left hemispheres were greater than those in the right hemispheres. Interestingly, in SCH FPCs, such asymmetries were almost completely reversed (Figure 5). In BPD FPCs, in contrast, such asymmetries were somewhat less obvious, differing somewhat from those of SCH in the densities of 7-AAD(+) and NeuN(+) nuclei (Figure 5).

There is a body of anatomical evidence suggesting that SCH is associated with disturbances in cerebral asymmetry [31]. Based largely on neuroimaging studies, the reported abnormalities in hemispheric asymmetry in SCH include a lack of normal asymmetry [32], less than normal asymmetry [33], and a reversal of normal asymmetry [34]-[36]. At the cellular level, however, the results remain mixed, partly due to various methodological problems associated with conventional histopathology as described above. Only Cullen et al. reported asymmetry anomalies in pyramidal cell densities and structures in the Nissl-stained sections of DLPFCs from SCH patients. Although they used conventional paraffin sections, tissue shrinkages during the embedding process were measured and matched between groups [37].

Although caution is still required when interpreting our data (e.g., the sample size was small when divided into hemispheres, and tissue from only one hemisphere was available from each subject in the present study), this is the first quantitative cell-counting report demonstrating the presence of left-greater-than-right asymmetries in the neural nuclei densities from the gray matter of control FPCs and their significant reversal in the gray matter of SCH FPCs. These findings are also strongly consistent with the above-mentioned gross anomalies that are typically found in SCH samples [31]-[36]. Considering the fact that most neuropathological studies have focused on only one (usually the left) hemisphere, future studies should utilize the brain collection in order to fully investigate the true laterality of the neural nuclei densities found in the human brain.

In conclusion, our FCM studies provide comprehensive evidence that these major psychiatric disorders do share similar neuropathological abnormalities. On the other hand, we also found certain neuropathological differences mainly in the FPC. If we take into account the fact that neurogenesis is almost exclusively restricted to early development [38], our findings suggest that the disturbances in NeuN(+) nuclei commonly observed in psychiatric disorders may occur over multiple stages of early brain development. In addition, disturbances in adulthood cortical myelination may also play a role in the origin of mood disorders. These disturbances may be influenced, to different extents, by genetic and/or environmental factors. Our innovative method could thus serve as a useful tool for future research, not only for the enhanced quantitative determinations of glial and interneuron subtypes in the human brain, but also for the study of animal models under varying degrees and conditions of genetic and environmental stress.

## Materials and Methods

All experimental protocols were approved by the Ethics Committee of the Tokyo Metropolitan Institute of Medical Science.

### Postmortem human brains

Frozen brain blocks from the FPC (BA10) and ITC (BA20) were generously provided by the Stanley Foundation Brain Collection (The Stanley Medical Research Institute, Bethesda, MD). Brain blocks were dissected from the lateral aspect of the frontal pole and from the gyral surface of the inferior temporal gyrus as described in the original paper by Brodmann [39]. The entire gray matter, from the surface to the border between layer VI and the white matter, was carefully dissected out manually from the frozen blocks with a sharp blade. About 20 mg of gray matter was collected from each specimen. The original collection consisted of 15 control, 15 BPD, and 15 SCH brains. Psychiatric diagnoses had been established by two psychiatrists using DSM-IV, medical records evaluations, and telephone interviews with family members, and were further examined by the Stanley Foundation review committee. We excluded 11 FPC and 11 ITC samples because they lacked entire gray matter layers as a result of previous experiments. We then analyzed the remaining samples (FPC, 12 control, 12 BPD, 10 SCH; ITC, 12 control, 11 BPD, 11 SCH). Demographic data on the brains included in this analysis are provided in Table S1. Ten out of 12 BPD subjects (FPC samples) and 8 out of 11 BPD subjects (ITC samples) had exhibited psychotic behavior. Other information about these brains has been described previously [40].

In all cases, the Stanley Medical Research Institute obtains written next-of-kin permission prior to using brains for research purposes. A detailed description of the Stanley Brain Collection can be obtained from the Institute's website at www.stanleyresearch.org.

### Nuclei isolation and FCM measurements

Tissue samples were processed and the counting, immunostaining, and FCM measurements of nuclei were performed as described by Hayashi et al. [16]. Briefly, a crude suspension of nuclei was obtained through mechanical homogenization of the brain tissue (1 min) in a 10-fold volume of lysis buffer (V/W) (0.32 M sucrose, 5 mM CaCl_2_, 3 mM magnesium acetate, 0.1 mM EDTA, 10 mM Tris-HCl [pH 8.0], 0.1% Triton X-100, osmolality ∼375 mOsm) using a set of plastic homogenizers. We chose this lysis buffer and homogenizers because Herculano-Houzel and Lent [41] had reported that when utilizing their isotropic fractionator method they were unable to obtain intact nuclei from unfixed brains (see Figure 1 in ref. [41]), which probably stemmed the use of an unsuitable lysis buffer with relatively high hypo-osmotic pressure (approximately 80 mOsm). The dissolved nuclei were centrifuged and then quickly suspended in PBS (280 mOsm) containing the fluorescent DNA marker 7-AAD (BD Pharmingen, Franklin Lakes, NJ). From this point on, nuclei were continuously suspended in solutions of 280 – 290 mOsm until FCM measurement in order to preserve nuclear size and structure. An aliquot of the 7-AAD stained nuclei was mixed with an equal amount of Flow-Count® (Beckman Coulter, Fullerton, CA) prior to calculating the total nuclear number by FCM (Epix XL, Beckman Coulter, Fullerton, CA). IsoFlowtm (290 mOsm) (Beckman Coulter, Fullerton, CA) was used as the sheath fluid. Once the total nuclei number was known, the proportion of neuronal and oligodendroglial nuclei was determined by immunocytochemical detection of the neuronal nuclear marker NeuN (Chemicon, Temecula, CA) and by the pan-oligodendroglial cell marker olig2 (Chemicon), respectively. Each absolute nuclear density was calculated by multiplying the number of total nuclei by each nuclear proportion. To investigate the stability of isolated nuclei, FCM measurements were performed 4 h or 18 h after homogenization and the resulting FS values for each nuclei were then compared.

### A comparison of human NeuN(+) nuclei size using visual and FCM measurements

The human samples (n = 2) were homogenized and then stained with NeuN and 7-AAD. The stained nuclei in PBS (280 mOsm) were dropped onto glass slides, overlaid with coverslips, and then observed under a fluorescent microscope (BX51; Olympus, Tokyo, Japan). Individual nuclear areas positive for NeuN or 7-AAD were traced and acquired with Image J software (available at http://rsb.info.nih.gov/nih-image). A total of two hundred NeuN(+) and 7-AAD(+) neuronal nuclei profiles were measured and analyzed. The same samples were also analyzed with FCM. The distribution of NeuN(+) nuclei FS values was plotted and compared with those of the 7-AAD(+) and NeuN(+) nuclei areas obtained by microscopic measurements.

### Animal studies

All experimental animal protocols were approved by the Animal Use and Care Committee of the Tokyo Metropolitan Institute of Medical Science.

Wistar rats (Oriental Yeast Co., Ltd., Tokyo, Japan) were maintained in a temperature-controlled room (∼23°C) with a 12 h/12 h light/dark cycle, in accordance with the Animal Use and Care Committee guidelines of the Tokyo Metropolitan Institute of Medical science. Experiments were performed during the light period. Eight-week-old rats (male, n = 8; female, n = 4) were killed with a lethal dose of anesthesia (pentobarbital sodium), and were then perfused with phosphate-buffered saline (PBS) containing 50 IU/ml heparin. The brains were then removed from the skull, and the cerebral cortex was dissected out as described by Herculano-Houzel and Lent [41]. The brains were immediately frozen and stored at −80°C until measurements were carried out. The frozen brains were used within a month, except for a group of brains from 8-week-old rats (n = 4), which were stored for 385 days in order to investigate the effects of frozen storage.

To determine the effects of postmortem intervals (PMIs), rats (8-week-old, male, n = 4) were killed by either a lethal dose of anesthesia or by cervical dislocation. The animals were then kept at 4°C for 0, 24, or 48 h without perfusion. At each PMI, brains were removed from the skull, the cerebral cortex was dissected out as described above, and the tissue was immediately frozen and stored at −80°C until measurements were conducted. These samples differed from the perfused samples in that they contained blood cells.

Each brain region was homogenized and their nuclei content measured as described above. Gender, frozen storage days, and PMIs had no significant effects on the absolute numbers of nuclei in the whole rat cerebral cortex (Figure S3).

For a comparison of the FS distributions of NeuN(+) nuclei, we chose two FS ranges (FS101-FS199 and FS251-FS349) and performed an unpaired *t*-test.

### Statistical analysis

Statistical analyses were carried out using GraphPad PRISM™ software (Graph Pad software, Inc., San Diego, CA) and SPSS™ (SPSS, Inc., Chicago, IL). To compare the numbers of nuclei in the psychiatric and control groups, we used an unpaired *t*-test. In order to determine whether countable confounding factors contributed significantly to the variances in the FCM measurements, we first performed Pearson’s correlations between the nuclei density of each cell type and the following factors: age at death (years), refrigeration interval (h), PMI (h), pH, weight (gram), and duration of frozen storage. Age at onset (years), duration of disease (years), Fluphenazine equivalent (mg) were studied separately due to a lack of control variables. The nuclei density was then compared between groups using analysis of covariance (ANCOVA) for any variables that contributed a significant proportion of variance to the FCM measurements. The effects of non-continuous confounding variables (gender, hemisphere, and severity of alcohol and/or substance abuse) on nuclei density were assessed using an unpaired *t*-test. To analyze size differences in the NeuN(+) nuclei, we divided the NeuN(+) nuclei population into subpopulations with forward scatter (FS) values (n ∼ n+99, where n ranged from 100 to 300 at intervals of 10) and analyzed all of the subpopulations with an unpaired *t*-test. In order to match certain confounding factors, such as the refrigeration interval and PMI between groups, we excluded samples with extremely long refrigeration intervals (> 20 h) and PMIs (> 40 h), and then reanalyzed the rest of the samples vis-à-vis the nuclei densities and sizes. When analyzing brain asymmetry, we used two-factor factorial ANOVA (diagnostic group×brain hemisphere).

## Supporting Information

Figure S1 **Microscopic measurements of isolated nuclei size.** (A) 7-AAD(+), (B) NeuN(+) nuclei in PBS (280 mOsm) prepared from unfixed frozen human cortical tissue. (C) A merged image. Scale bar, 25 µm. (D) A traced image of 7-AAD(+) nuclei in panel A by Image J. (E) A traced image of NeuN(+) nuclei in panel A by Image J.
(TIF)

Figure S2 **Effects of confounding factors on the FS distribution of NeuN(+) nuclei from rat cerebral cortices.** (A) Effect of gender. No significant difference was found in the FS distribution of NeuN(+) nuclei between males (blue) and females (red) (unpaired *t*-test, FS100-199, *t*(6) = -1.081, *P* = 0.321; FS250-349, *t*(6) = 1.134, *P* = 0.300). (B) Effect of frozen storage. No significant difference was found in the FS distribution of NeuN(+) nuclei at 20 (blue) or 385 days (red) (unpaired *t*-test, FS100-199, *t*(6) = -0.265, *P* = 0.800; FS250-349, *t*(6) = 0.498, *P* = 0.636). (C) Effect of PMI. Brains were dissected out at 0 (blue), 24 (red), and 48 (green) h after the rats were sacrificed. With increasing PMIs, the FS distribution peaks of the NeuN(+) nuclei shifted towards larger FS values. No significant difference was found in small NeuN(+) nuclei (unpaired *t*-test, FS100-199, F(2,9) = 3.132, *P* = 0.093), while a significant difference in the large NeuN(+) nuclei was noted (FS250-349, F(2,9) = 13.441, *P* = 0.002).
(TIF)

Figure S3 **Effects of confounding factors on the absolute nuclear numbers in the whole rat cerebral hemisphere.** Total (blue), NeuN (red), olig2 (green), and NeuN(−)/olig2(−) (purple) nuclei numbers in the whole rat cerebral hemisphere (×10^6^ cells/brain, 8 month-old) are shown. (A) Effect of PMI. PMI exerted no significant effect on any of the nuclei numbers (one-way ANOVA, total, F(2,9) = 0.48, *P* = 0.633; NeuN(+), F(2,9) = 0.98, *P* = 0.412; olig2(+), F(2,9) = 0.33, *P* = 0.727; NeuN(−)/olig2(−), F(2,9) = 1.99, *P* = 0.193). (B) Effect of frozen storage. Duration of storage (days) had no significant effect on any of the nuclei numbers (unpaired *t*-test, total, *t*(6) = -0.094, *P* = 0.928; NeuN(+), *t*(6) = 0.140, *P* = 0.893; olig2(+); *t*(6) = -1.764, *P* = 0.128; NeuN(−)/olig2(−); *t*(6) = 1.106, *P* = 0.311). Data represent mean±s.d. Note that these findings suggest that neither PMIs nor frozen storage reduce the immunoreactivities of NeuN or olig2 to any significant degree.
(TIF)

Table S1 Demographic data of individuals from whom FPC and ITC samples were obtained.
(DOC)

Table S2 Statistical results of the FS distribution of NeuN(+) nuclei by unpaired *t*-test.
(DOC)

Table S3 Correlation between each nuclei number and the confounding factors (Part I).
(DOC)

Table S4 Correlation between each nuclei number and the confounding factors (Part II).
(DOC)

Table S5 Statistical results of nuclei densities in the FPC or ITC from the selected subjects excluding those with longer refrigeration intervals (> 20 h) and PMIs (> 40 h).
(DOC)

Table S6 Statistical results of the FS distribution of NeuN(+) nuclei in the FPC or ITC from the selected subjects, excluding those with longer refrigeration intervals (> 20 h) and PMIs (> 40 h).
(DOC)

Table S7 Effects of gender, hemisphere, and substance and/or alcohol abuse on each nuclei density in FPC gray matter.
(DOC)

Table S8 Effects of gender, hemisphere, and substance and/or alcohol abuse on each nuclei density in ITC gray matter.
(DOC)
