# Supplementary figures and images for: Neuropathological Similarities and Differences between Schizophrenia and Bipolar Disorder: A Flow Cytometric Postmortem Brain Study
Source: PLoS One. 2012 Mar 15;7(3):e33019. doi: 10.1371/journal.pone.0033019 (PMC3305297; doi:10.1371/journal.pone.0033019)

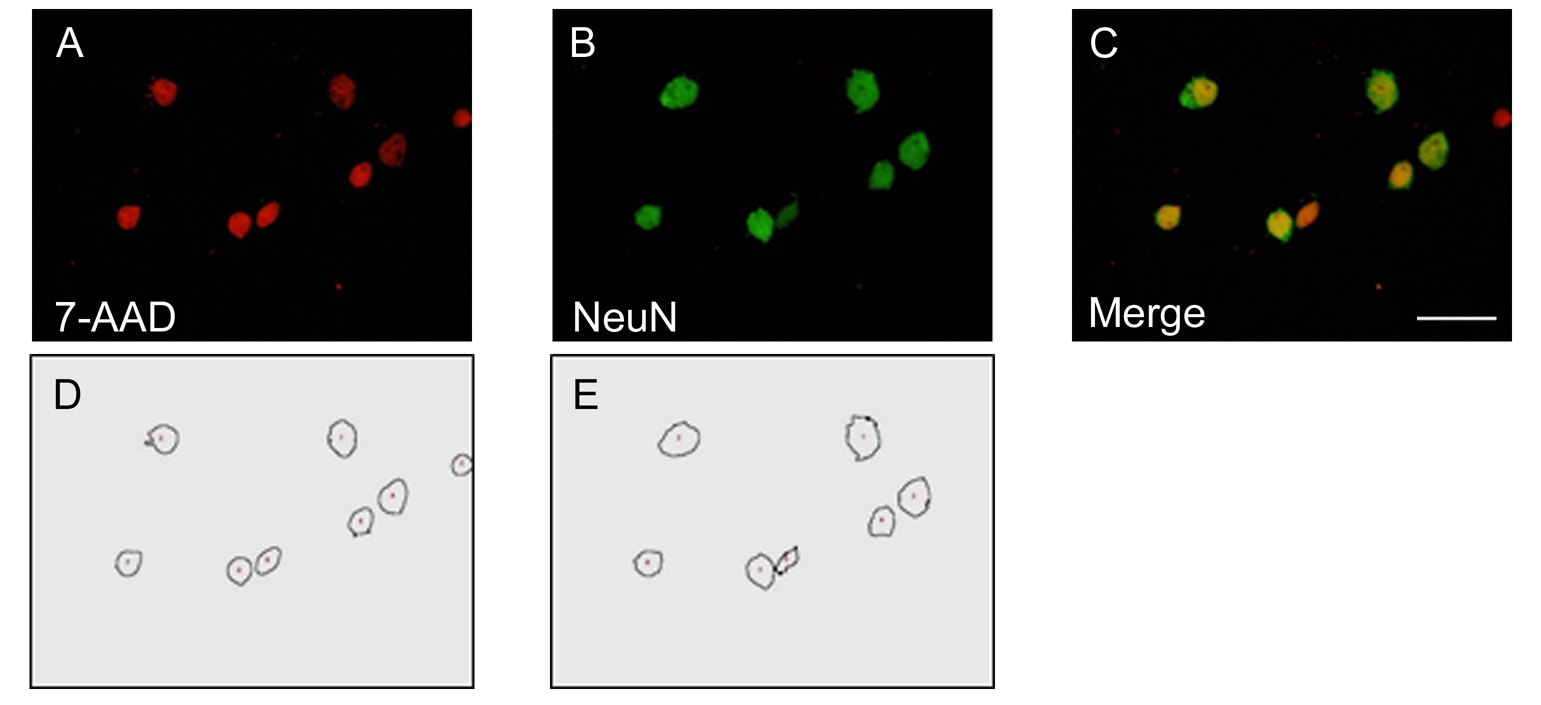

Supplement: Figure S1 — Microscopic measurements of isolated nuclei size. (A) 7-AAD(+), (B) NeuN(+) nuclei in PBS (280 mOsm) prepared from unfixed frozen human cortical tissue. (C) A merged image. Scale bar, 25 µm. (D) A traced image of 7-AAD(+) nuclei in panel A by Image J. (E) A traced image of NeuN(+) nuclei in panel A by Image J. (TIF) [file pone.0033019.s001.tif]

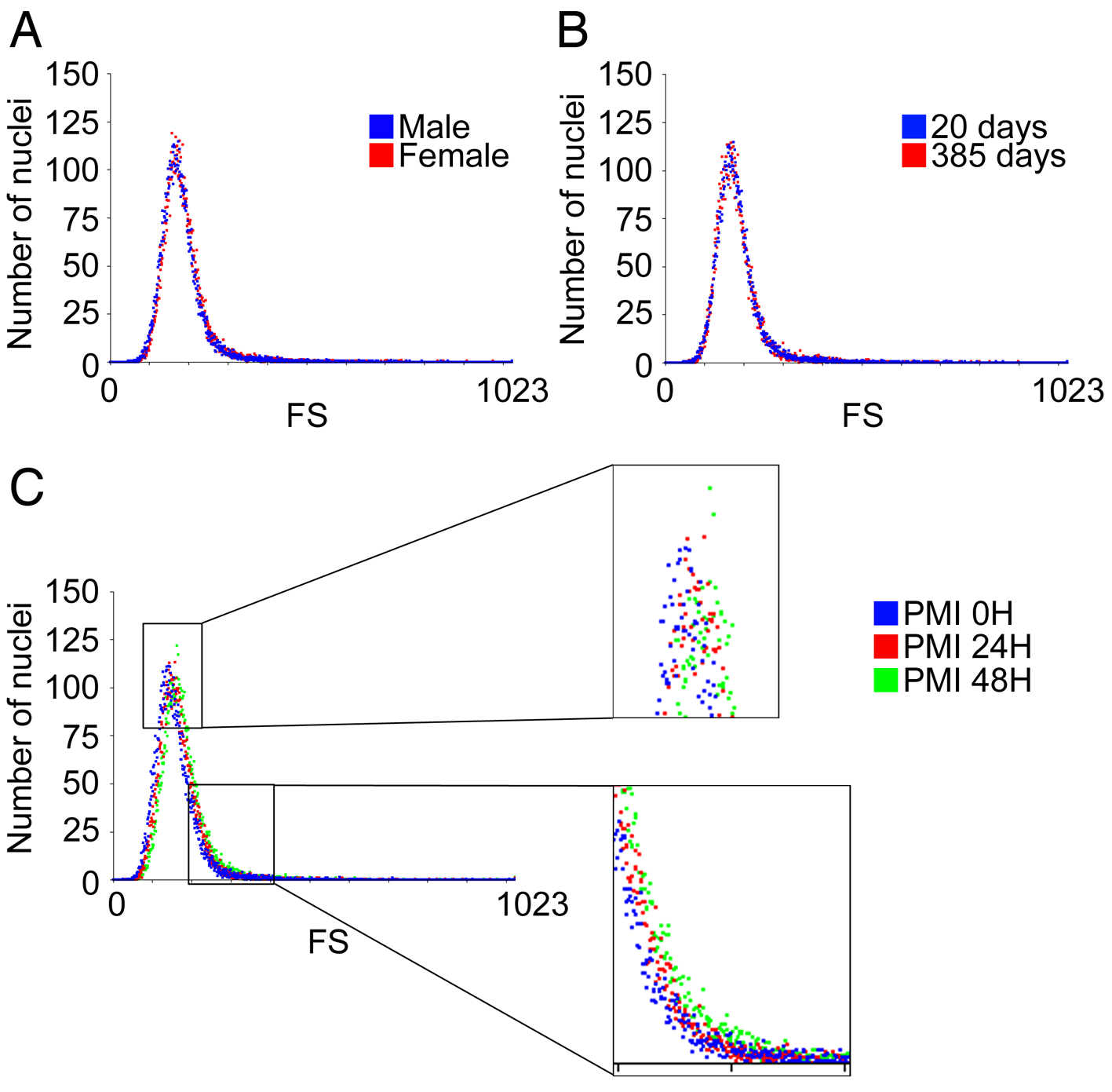

Supplement: Figure S2 — Effects of confounding factors on the FS distribution of NeuN(+) nuclei from rat cerebral cortices. (A) Effect of gender. No significant difference was found in the FS distribution of NeuN(+) nuclei between males (blue) and females (red) (unpaired t-test, FS100-199, t(6) = -1.081, P = 0.321; FS250-349, t(6) = 1.134, P = 0.300). (B) Effect of frozen storage. No significant difference was found in the FS distribution of NeuN(+) nuclei at 20 (blue) or 385 days (red) (unpaired t-test, FS100-199, t(6) = -0.265, P = 0.800; FS250-349, t(6) = 0.498, P = 0.636). (C) Effect of PMI. Brains were dissected out at 0 (blue), 24 (red), and 48 (green) h after the rats were sacrificed. With increasing PMIs, the FS distribution peaks of the NeuN(+) nuclei shifted towards larger FS values. No significant difference was found in small NeuN(+) nuclei (unpaired t-test, FS100-199, F(2,9) = 3.132, P = 0.093), while a significant difference in the large NeuN(+) nuclei was noted (FS250-349, F(2,9) = 13.441, P = 0.002). (TIF) [file pone.0033019.s002.tif]

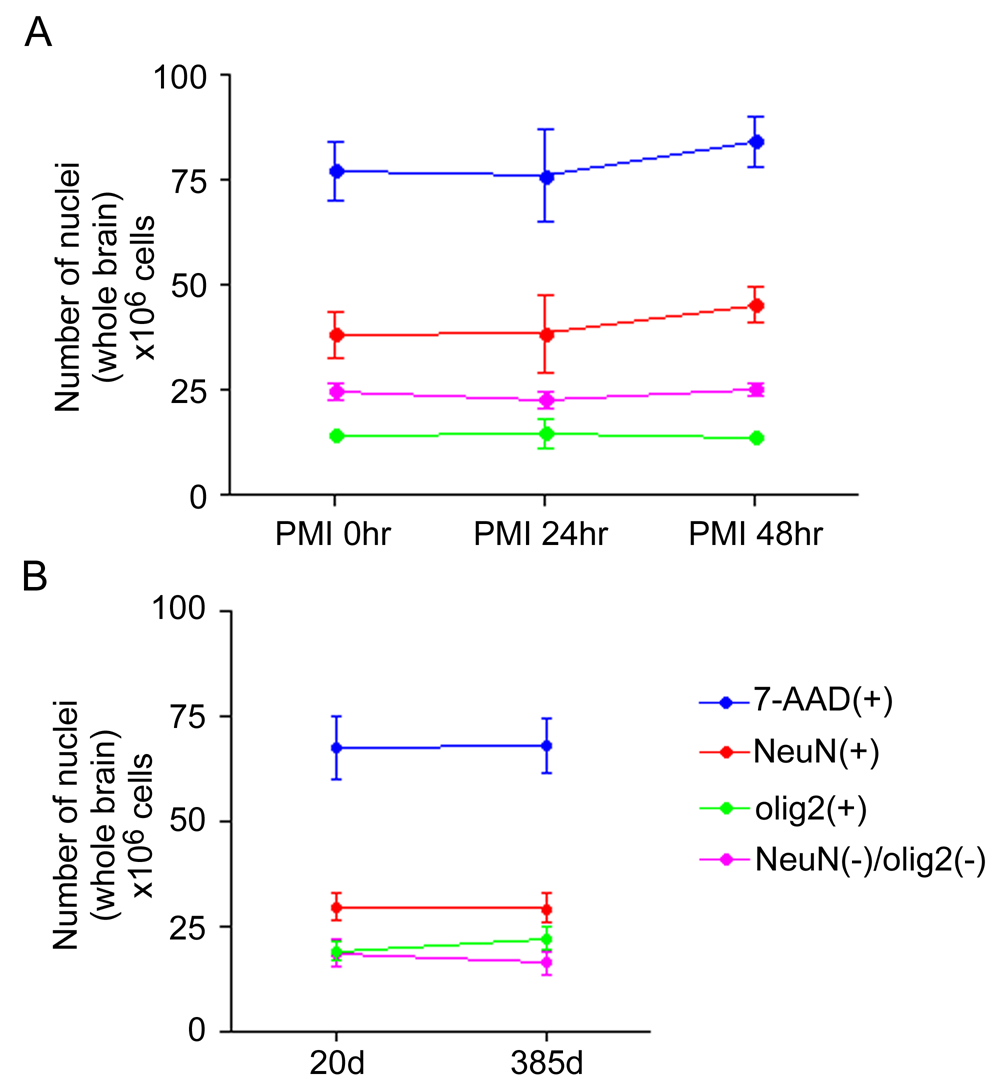

Supplement: Figure S3 — Effects of confounding factors on the absolute nuclear numbers in the whole rat cerebral hemisphere. Total (blue), NeuN (red), olig2 (green), and NeuN(−)/olig2(−) (purple) nuclei numbers in the whole rat cerebral hemisphere (×106 cells/brain, 8 month-old) are shown. (A) Effect of PMI. PMI exerted no significant effect on any of the nuclei numbers (one-way ANOVA, total, F(2,9) = 0.48, P = 0.633; NeuN(+), F(2,9) = 0.98, P = 0.412; olig2(+), F(2,9) = 0.33, P = 0.727; NeuN(−)/olig2(−), F(2,9) = 1.99, P = 0.193). (B) Effect of frozen storage. Duration of storage (days) had no significant effect on any of the nuclei numbers (unpaired t-test, total, t(6) = -0.094, P = 0.928; NeuN(+), t(6) = 0.140, P = 0.893; olig2(+); t(6) = -1.764, P = 0.128; NeuN(−)/olig2(−); t(6) = 1.106, P = 0.311). Data represent mean±s.d. Note that these findings suggest that neither PMIs nor frozen storage reduce the immunoreactivities of NeuN or olig2 to any significant degree. (TIF) [file pone.0033019.s003.tif]
