# Supplementary material for: Neuropathological Similarities and Differences between Schizophrenia and Bipolar Disorder: A Flow Cytometric Postmortem Brain Study
Source: PLoS One. 2012 Mar 15;7(3):e33019. doi: 10.1371/journal.pone.0033019 (PMC3305297; doi:10.1371/journal.pone.0033019)
Supplement: Table S1 — Demographic data of individuals from whom FPC and ITC samples were obtained. (DOC) [file pone.0033019.s004.doc]

| Parameter | |  | Normal control |  | Bipolar disorder |  | Schizophrenia |  | Control *vs.* BPD | | |  | Control *vs.* SCH | | |  |
| --- | --- | --- | --- | --- | --- | --- | --- | --- | --- | --- | --- | --- | --- | --- | --- | --- |
|  |  |  |  |  |  |  |  |  | *t* | *df** | *P* value |  | *t* | *df** | *P* value | Technique |
| **Frontopolar cortex** | |  |  |  |  |  |  |  |  |  |  |  |  |  |  |  |
|  | *n* |  | 12 |  | 12 |  | 10 |  |  |  |  |  |  |  |  |  |
|  | Age at death (years) |  | 48 (11) [29-68] |  | 41 (11) [25-57] |  | 43 (14) [25-62] |  | 1.78 | 22 | 0.089 |  | 1.05 | 20 | 0.306 | Unpaired t test |
|  | Gender |  | 8M/4F |  | 7M/5F |  | 7M/3F |  | NA | 1 | 1.000 |  | NA | 1 | 1.000 | Fisher's exact probability test |
|  | Refrigeration interval (hour) |  | 3.5 (1.5) [1-6] |  | 7.5 (6.3) [2-21] |  | 10.1 (8.4) [3-27] |  | -2.15 | 12.27 | 0.052 |  | -2.46 | 9.49 | 0.035 | Unpaired t test |
|  | PMI (hour) |  | 23.4 (11.2) [8-42] |  | 27.8 (13.5) [13-62] |  | 38.6 (14.1) [19-61] |  | -0.86 | 22 | 0.402 |  | -2.82 | 20 | 0.011 | Unpaired t test |
|  | Brain pH |  | 6.3 (0.2) [5.8-6.6] |  | 6.2 (0.2) [5.8-6.5] |  | 6.2 (0.2) [5.8-6.6] |  | 0.48 | 22 | 0.634 |  | 0.37 | 20 | 0.717 | Unpaired t test |
|  | Side of Brain |  | 5R/7L |  | 6R/6L |  | 4R/6L |  | NA | 1 | 1.000 |  | NA | 1 | 1.000 | Fisher's exact probability test |
|  | Brain weight |  | 1492 (157) [1305-1840] |  | 1411 (170) [1130-1680] |  | 1475 (129) [1270-1640] |  | 1.21 | 22 | 0.239 |  | 0.28 | 20 | 0.786 | Unpaired t test |
|  | Storage days |  | 4249 (232) [3945-4683] |  | 4530 (184) [4151-4763] |  | 4632 (176) [4334-4858] |  | -3.29 | 22 | 0.003 |  | -4.28 | 20 | <0.001 | Unpaired t test |
|  | Age at onset (years) |  | - |  | 21.5 (6.4) [14-34] |  | 21.8 (7.3) [13-38] |  | - | - | - |  | - | - | - |  |
|  | Duration of disease (years) |  | - |  | 18.5 (8.0) [6-32] |  | 21.2 (13.4) [5-45] |  | - | - | - |  | - | - | - |  |
|  | History of psychosis |  | - |  | 10 with / 2 without |  | 10 with |  | - | - | - |  | - | - | - |  |
|  | Fluphenazine equivalent (mg) |  | - |  | 22492 (25053) [0-60000] |  | 26500 (28356) [0-80000] |  | - | - | - |  | - | - | - |  |
|  | History of alcohol and/or drug use† | | |  |  |  |  |  |  |  |  |  |  |  |  |  |
|  | Never |  | 10 |  | 4 |  | 6 |  |  |  |  |  |  |  |  |  |
|  | Current |  | 0 |  | 6 |  | 3 |  | NA | 2 | 0.014 |  | NA | 2 | 0.123 | χ^2^ test for independence |
|  | Past |  | 2 |  | 2 |  | 1 |  |  |  |  |  |  |  |  |  |
|  | Smoking at time of death |  |  |  |  |  |  |  |  |  |  |  |  |  |  |  |
|  | Yes |  | 2 |  | 6 |  | 4 |  |  |  |  |  |  |  |  |  |
|  | No |  | 4 |  | 3 |  | 3 |  | NA | 1 | 0.315 |  | NA | 1 | 0.592 | Fisher's exact probability test |
|  | (Unknown) |  | 6 |  | 3 |  | 3 |  |  |  |  |  |  |  |  |  |
| **Inferior temporal cortex** | |  |  |  |  |  |  |  |  |  |  |  |  |  |  |  |
|  | *n* |  | 12 |  | 11 |  | 11 |  |  |  |  |  |  |  |  |  |
|  | Age at death (years) |  | 50 (10) [35-68] |  | 41 (12) [25-61] |  | 45 (14) [25-62] |  | 1.95 | 21 | 0.065 |  | 0.96 | 18.055 | 0.349 | Unpaired t test |
|  | Gender |  | 7M/5F |  | 6M/5F |  | 6M/5F |  | NA | 1 | 1.000 |  | NA | 1 | 1.000 | Fisher's exact probability test |
|  | Refrigeration interval (hour)** |  | 3.6 (1.5) [1-6] |  | 10.8 (10.9) [3-39] |  | 9.8 (8.0) [3-27] |  | -2.18 | 10.35 | 0.053 |  | -2.55 | 10.65 | 0.028 | Unpaired t test |
|  | PMI (hour) |  | 23.5 (8.8) [8-40] |  | 32.7 (15.4) [13-60] |  | 35.1 (14.9) [19-61] |  | -1.78 | 15.629 | 0.102 |  | -2.29 | 21 | 0.033 | Unpaired t test |
|  | Brain pH |  | 6.3 (0.3) [5.8-6.6] |  | 6.2 (0.3) [5.8-6.5] |  | 6.2 (0.2) [5.8-6.6] |  | 1.00 | 21 | 0.329 |  | 0.56 | 21 | 0.585 | Unpaired t test |
|  | Side of Brain |  | 6R/6L |  | 7R/4L |  | 4R/7L |  | NA | 1 | 0.680 |  | NA | 1 | 0.680 | Fisher's exact probability test |
|  | Brain weight |  | 1520 (176) [1305-1840] |  | 1436 (183) [1130-1690] |  | 1451 (109) [1270-1620] |  | 1.11 | 21 | 0.278 |  | 1.10 | 21 | 0.285 | Unpaired t test |
|  | Storage days |  | 4775 (247) [4459-5189] |  | 5103 (114) [4937-5256] |  | 5067 (260) [4496-5344] |  | -4.03 | 15.726 | 0.001 |  | -2.77 | 21 | 0.011 | Unpaired t test |
|  | Age at onset (years) |  | - |  | 20.8 (9.3) [7-39] |  | 22.7 (6.8) [13-38] |  | - | - | - |  | - | - | - |  |
|  | Duration of disease (years) |  | - |  | 19.7 (10.6) [6-43] |  | 21.9 (13.0) [5-45] |  | - | - | - |  | - | - | - |  |
|  | History of psychosis |  | - |  | 8 with / 3 without |  | 11 with |  | - | - | - |  | - | - | - |  |
|  | Fluphenazine equivalent (mg) |  | - |  | 14764 (20207) [0-60000] |  | 55818 (65181) [0-200000] |  | - | - | - |  | - | - | - |  |
|  | History of alcohol and/or drug use† | | |  |  |  |  |  |  |  |  |  |  |  |  |  |
|  | Never |  | 10 |  | 4 |  | 7 |  |  |  |  |  |  |  |  |  |
|  | Current |  | 0 |  | 5 |  | 3 |  | NA | 2 | 0.023 |  | NA | 2 | 0.148 | χ^2^ test for independence |
|  | Past |  | 2 |  | 2 |  | 1 |  |  |  |  |  |  |  |  |  |
|  | Smoking at time of death‡ |  |  |  |  |  |  |  |  |  |  |  |  |  |  |  |
|  | Yes |  | 2 |  | 5 |  | 5 |  |  |  |  |  |  |  |  |  |
|  | No |  | 4 |  | 2 |  | 3 |  | NA | 1 | 0.286 |  | NA | 1 | 0.592 | Fisher's exact probability test |
|  | (Unknown) |  | 6 |  | 4 |  | 3 |  |  |  |  |  |  |  |  |  |

Values are expressed as mean (SD) [range]; numbers in brackets indicate minimum and maximum values. M, male; F, female; PMI, post-mortem interval in hours; R, right; L, left; FPC, frontopolar cortex; ITC, inferior temporal cortex

*The *dfs* differ among variables owing to the unavailability of data for some specimens.

**One set of control data was unavailable.

†History of alcohol and/or drug use: *never*, no history of use; *current*, drug and/or alcohol use at the time of death; *past*, a history of alcohol or drug use, though not in the 6 months prior to death.

‡Group difference for “Smoking at time of death” was analyzed without including any unknown subjects
