# Supplementary material for: Neuropathological Similarities and Differences between Schizophrenia and Bipolar Disorder: A Flow Cytometric Postmortem Brain Study
Source: PLoS One. 2012 Mar 15;7(3):e33019. doi: 10.1371/journal.pone.0033019 (PMC3305297; doi:10.1371/journal.pone.0033019)
Supplement: Table S2 — Statistical results of the FS distribution of NeuN(+) nuclei by unpaired t-test. (DOC) [file pone.0033019.s005.doc]

FS, forward scatter; Cont, normal control; BPD, bipolar disorder; SCH, schizophrenia. The data of FS200-299 (yellow) are shown in Figure 4. **P*<0.05 by unpaired *t*-test.
