# Supplementary material for: Neuropathological Similarities and Differences between Schizophrenia and Bipolar Disorder: A Flow Cytometric Postmortem Brain Study
Source: PLoS One. 2012 Mar 15;7(3):e33019. doi: 10.1371/journal.pone.0033019 (PMC3305297; doi:10.1371/journal.pone.0033019)
Supplement: Table S3 — Correlation between each nuclei number and the confounding factors (Part I). (DOC) [file pone.0033019.s006.doc]

Pearson’s correlation coefficient (*r*) values, *P* values, and sample numbers (*n*) are given.

* In the postmortem brain from the ITC of a normal control, one sample lacked any refrigeration interval.

Significant positive correlations (*P*<0.05) are highlighted in yellow.
