# Supplementary material for: Neuropathological Similarities and Differences between Schizophrenia and Bipolar Disorder: A Flow Cytometric Postmortem Brain Study
Source: PLoS One. 2012 Mar 15;7(3):e33019. doi: 10.1371/journal.pone.0033019 (PMC3305297; doi:10.1371/journal.pone.0033019)
Supplement: Table S4 — Correlation between each nuclei number and the confounding factors (Part II). (DOC) [file pone.0033019.s007.doc]

Pearson’s correlation coefficient (*r*) values, *P* values, and sample numbers (*n*) are given.

Significant positive correlations (*P*<0.05) are highlighted in yellow.

Note: ANCOVA further revealed that PMI made no impact on our main findings in terms of the number of total, NeuN(+), or NeuN(-)/olig2(-) nuclei when comparing BPD and SCH (total, *P* = 0.943; NeuN(+), *P* = 0.988; NeuN(-)/olig2(-), *P* = 0.245).
