# Supplementary material for: Neuropathological Similarities and Differences between Schizophrenia and Bipolar Disorder: A Flow Cytometric Postmortem Brain Study
Source: PLoS One. 2012 Mar 15;7(3):e33019. doi: 10.1371/journal.pone.0033019 (PMC3305297; doi:10.1371/journal.pone.0033019)
Supplement: Table S5 — Statistical results of nuclei densities in the FPC or ITC from the selected subjects excluding those with longer refrigeration intervals (> 20 h) and PMIs (> 40 h). (DOC) [file pone.0033019.s008.doc]

Statistical analyses were performed by unpaired *t*-test. mean (SD).

Among the selected samples, no statistically significant difference was found in the refrigeration interval (FPC, Cont *vs.* BPD, *t*(10.8)=-1.95, *P*=0.078; Cont vs. SCH, *t*(4.31)=-1.77, *P*=0.146; ITC, Cont *vs.* BPD, *t*(8.97)=-1.62, *P*=0.140; Cont vs. SCH, *t*(7.03)=-2.14, *P*=0.070) and the PMI (FPC, Cont *vs.* BPD, *t*(15.7)=-0.85, *P*=0.408; Cont vs. SCH, *t*(13)=-2.09, *P*=0.057; ITC, Cont *vs.* BPD, *t*(16)=-0.79, *P*=0.443; Cont vs. SCH, *t*(15)=-1.39, *P*=0.185).

Note that essentially the same results as those demonstrated in Figure 1 were obtained in the selected samples for both the FPC and ITC.
