# Supplementary material for: Neuropathological Similarities and Differences between Schizophrenia and Bipolar Disorder: A Flow Cytometric Postmortem Brain Study
Source: PLoS One. 2012 Mar 15;7(3):e33019. doi: 10.1371/journal.pone.0033019 (PMC3305297; doi:10.1371/journal.pone.0033019)
Supplement: Table S6 — Statistical results of the FS distribution of NeuN(+) nuclei in the FPC or ITC from the selected subjects, excluding those with longer refrigeration intervals (> 20 h) and PMIs (> 40 h). (DOC) [file pone.0033019.s009.doc]

FS, forward scatter; Cont, normal control; BPD, bipolar disorder; SCH, schizophrenia. **P*<0.05 by unpaired *t*-test

Note that essentially the same results as those demonstrated in eTable 5 were obtained in the selected samples.
