# Supplementary material for: Neuropathological Similarities and Differences between Schizophrenia and Bipolar Disorder: A Flow Cytometric Postmortem Brain Study
Source: PLoS One. 2012 Mar 15;7(3):e33019. doi: 10.1371/journal.pone.0033019 (PMC3305297; doi:10.1371/journal.pone.0033019)
Supplement: Table S8 — Effects of gender, hemisphere, and substance and/or alcohol abuse on each nuclei density in ITC gray matter. (DOC) [file pone.0033019.s011.doc]

Statistical analyses were performed by unpaired *t*-test (Control *vs.* BPD, Control *vs.* SCH). Values are expressed as mean (SD). ITC, inferior temporal cortex; BPD, bipolar disorder; SCH, schizophrenia.
